# A radiomics-based interpretable model to predict the pathological grade of pancreatic neuroendocrine tumors

**DOI:** 10.1007/s00330-023-10186-1

**Published:** 2023-09-02

**Authors:** Jing-Yuan Ye, Peng Fang, Zhen-Peng Peng, Xi-Tai Huang, Jin-Zhao Xie, Xiao-Yu Yin

**Affiliations:** 1 Department of Pancreato-Biliary Surgery, The First Affiliated Hospital of Sun Yat-Sen University, No.58 Zhongshan Er Road, Guangzhou, 510080 Guangdong People’s Republic of China; 2 Department of Radiology, The First Affiliated Hospital of Sun Yat-Sen University, No.58 Zhongshan Er Road, Guangzhou, Guangdong People’s Republic of China

**Keywords:** Radiomics, Machine learning, Interpretability, Pancreatic neuroendocrine tumors, Pathological grade

## Abstract

**Objectives:**

To develop a computed tomography (CT) radiomics-based interpretable machine learning (ML) model to predict the pathological grade of pancreatic neuroendocrine tumors (pNETs) in a non-invasive manner.

**Methods:**

Patients with pNETs who underwent contrast-enhanced abdominal CT between 2010 and 2022 were included in this retrospective study. Radiomics features were extracted, and five radiomics-based ML models, namely logistic regression (LR), random forest (RF), support vector machine (SVM), XGBoost, and GaussianNB, were developed. The performance of these models was evaluated using a time-independent testing set, and metrics such as sensitivity, specificity, accuracy, and the area under the receiver operating characteristic curve (AUC) were calculated. The accuracy of the radiomics model was compared to that of needle biopsy. The Shapley Additive Explanation (SHAP) tool and the correlation between radiomics and biological features were employed to explore the interpretability of the model.

**Results:**

A total of 122 patients (mean age: 50 ± 14 years; 53 male) were included in the training set, whereas 100 patients (mean age: 48 ± 13 years; 50 male) were included in the testing set. The AUCs for LR, SVM, RF, XGBoost, and GaussianNB were 0.758, 0.742, 0.779, 0.744, and 0.745, respectively, with corresponding accuracies of 73.0%, 70.0%, 77.0%, 71.9%, and 72.9%. The SHAP tool identified two features of the venous phase as the most significant, which showed significant differences among the Ki-67 index or mitotic count subgroups (*p* < 0.001).

**Conclusions:**

An interpretable radiomics-based RF model can effectively differentiate between G1 and G2/3 of pNETs, demonstrating favorable interpretability.

**Clinical relevance statement:**

The radiomics-based interpretable model developed in this study has significant clinical relevance as it offers a non-invasive method for assessing the pathological grade of pancreatic neuroendocrine tumors and holds promise as an important complementary tool to traditional tissue biopsy.

**Key Points:**

*• A radiomics-based interpretable model was developed to predict the pathological grade of pNETs and compared with preoperative needle biopsy in terms of accuracy.*

*• The model, based on CT radiomics, demonstrated favorable interpretability.*

*• The radiomics model holds potential as a valuable complementary technique to preoperative needle biopsy; however, it should not be considered a replacement for biopsy.*

**Supplementary Information:**

The online version contains supplementary material available at 10.1007/s00330-023-10186-1.

## Introduction

Pancreatic neuroendocrine tumors (pNETs) account for approximately 5% of all pancreatic tumors and originate from neuroendocrine cells of the pancreas, showing heterogeneous characteristics [1]. Recent studies have shown that the incidence of pNETs has increased steadily in the past two decades [2, 3]. The 2019 WHO classification classifies pNETs into two categories: well-differentiated neuroendocrine tumors and poorly differentiated neuroendocrine carcinomas, according to differentiation and cell proliferation activity. The well-defined tumors could be further categorized into G1, G2, and G3 based on the Ki-67 index and mitotic count [4]. The pathological grade of pNETs is strongly correlated with the prognosis [5–8]. The 5-year overall survival (OS) rates for G1, G2, and G3 tumors were reported as 77.33%, 63.06%, and 20.04%, respectively [3].

Histopathological evaluation plays a crucial role in determining the prognosis and tailoring the treatment of patients with pNETs. Depending on the extent of tumor spread, namely locoregional disease, locally advanced disease, or distant metastasis, the pathological grade of pNETs is particularly important for selecting appropriate initial treatment strategies [9–12]. Therefore, individual assessment of the pathological grade is typically needed before treatment. Among all currently available examination methods, endoscopic ultrasound-guided fine needle aspiration (EUS-FNA) biopsy is a valuable technique for obtaining the histological grade of the tumors [13]. However, this is an invasive procedure and requires at least 500 cells for grading, which sets a technical threshold for this procedure. Therefore, owing to insufficient tissue samples, only about 70% of patients undergoing EUS-FNA biopsy can obtain a histologic grade [14]. Furthermore, owing to the small size of the biopsy samples and the heterogeneity of pNETs, biopsy results are often inconsistent with postoperative pathology. A recent meta-analysis showed that the concordance rates between EUS-FNA/FNB grades and surgical specimens ranged from 53.8 to 97.1% [15].

Radiomics is an emerging technology that involves the extraction of quantitative and reproducible features from medical images in high-throughput, sophisticated modalities that are challenging to identify or quantify visually [16]. These features, which may be associated with specific diseases, are processed using statistical or machine learning (ML) algorithms to establish predictive models for tumor diagnosis, grading, efficacy evaluation, and prognosis prediction [17–19]. The main advantages of radiomics are its non-invasiveness, objectivity, and reproducibility. However, the limited sample sizes and the lack of interpretability of the ML-based models have restricted the application of radiomics-based studies in clinical practice.

This study aimed to develop a radiomics-based ML model that could predict the grade of pNETs in a non-invasive manner using CT images. In addition, the study aimed to explore the interpretability of the radiomics model by establishing the relationship between the radiomics features and biological features of the tumors.

## Materials and methods

### Patient identification

**Fig. 1:**
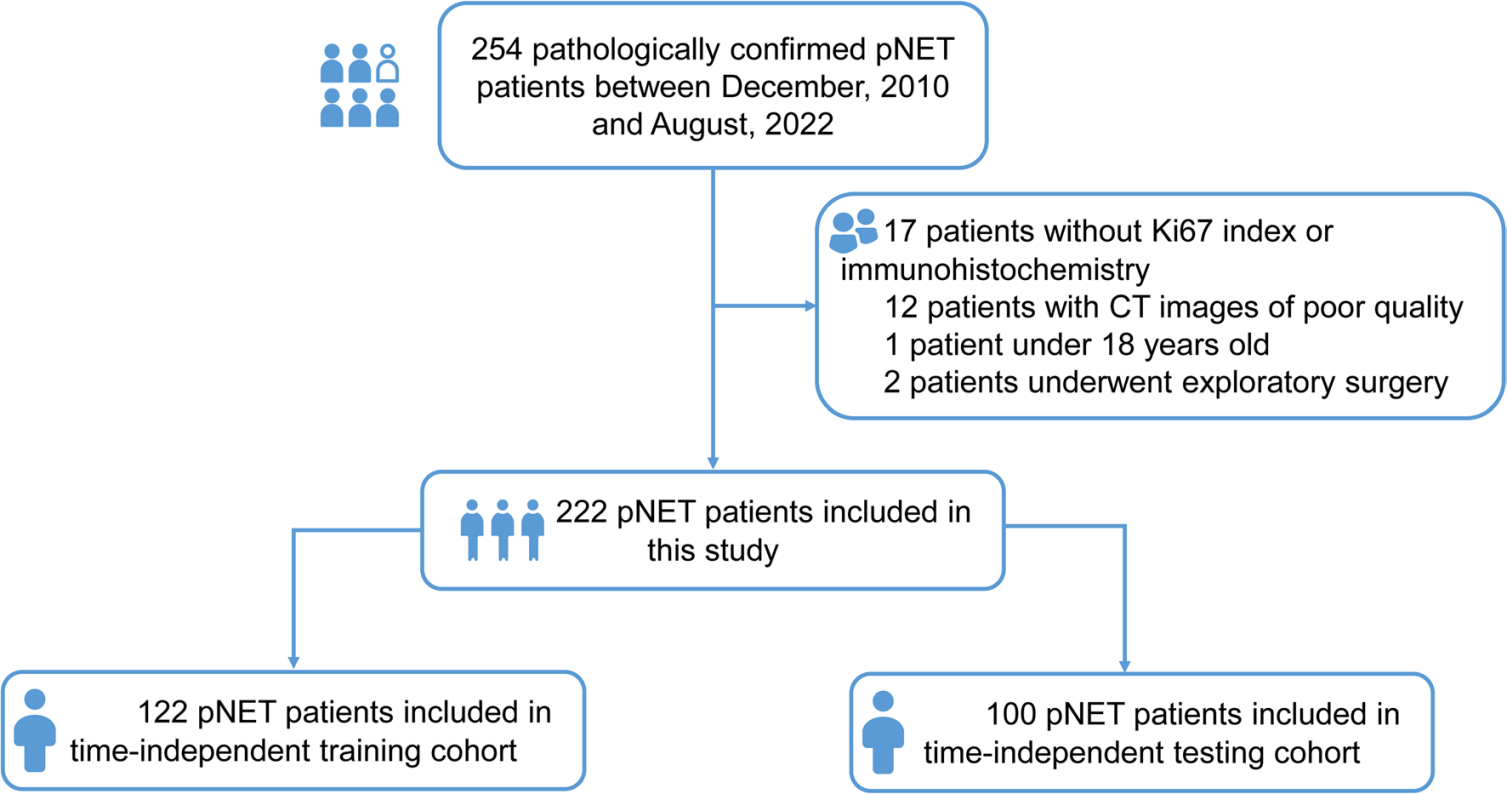
Flowchart of the study

This study retrospectively included patients with pathologically confirmed pNETs at the First Affiliated Hospital of Sun Yat-sen University from December 2010 to August 2022. As illustrated in Fig. 1, The inclusion criteria included the following: (1) histopathological diagnosis of pNETs by surgical specimen; (2) availability of contrast-enhanced abdominal CT within 1 month before surgery. The exclusion criteria included the following: (1) age less than 18 years; (2) absence of pathological grade information; (3) CT images with artifacts; (4) CT images without visible lesions; (5) less than 1 cm of long diameter of the lesion; (6) patients who only underwent exploratory laparotomy. The decision to undertake surgical intervention for patients was informed by the most current National Comprehensive Cancer Network (NCCN) guidelines [20–22]. This retrospective study was approved by the institutional review board, and the informed consent was waived.

The patients were split into a training set and a time-independent testing set. Patients between January 2019 and August 2022 constituted the training set, and patients between December 2010 and December 2018 formed the testing set.

### Workflow

**Fig. 2:**
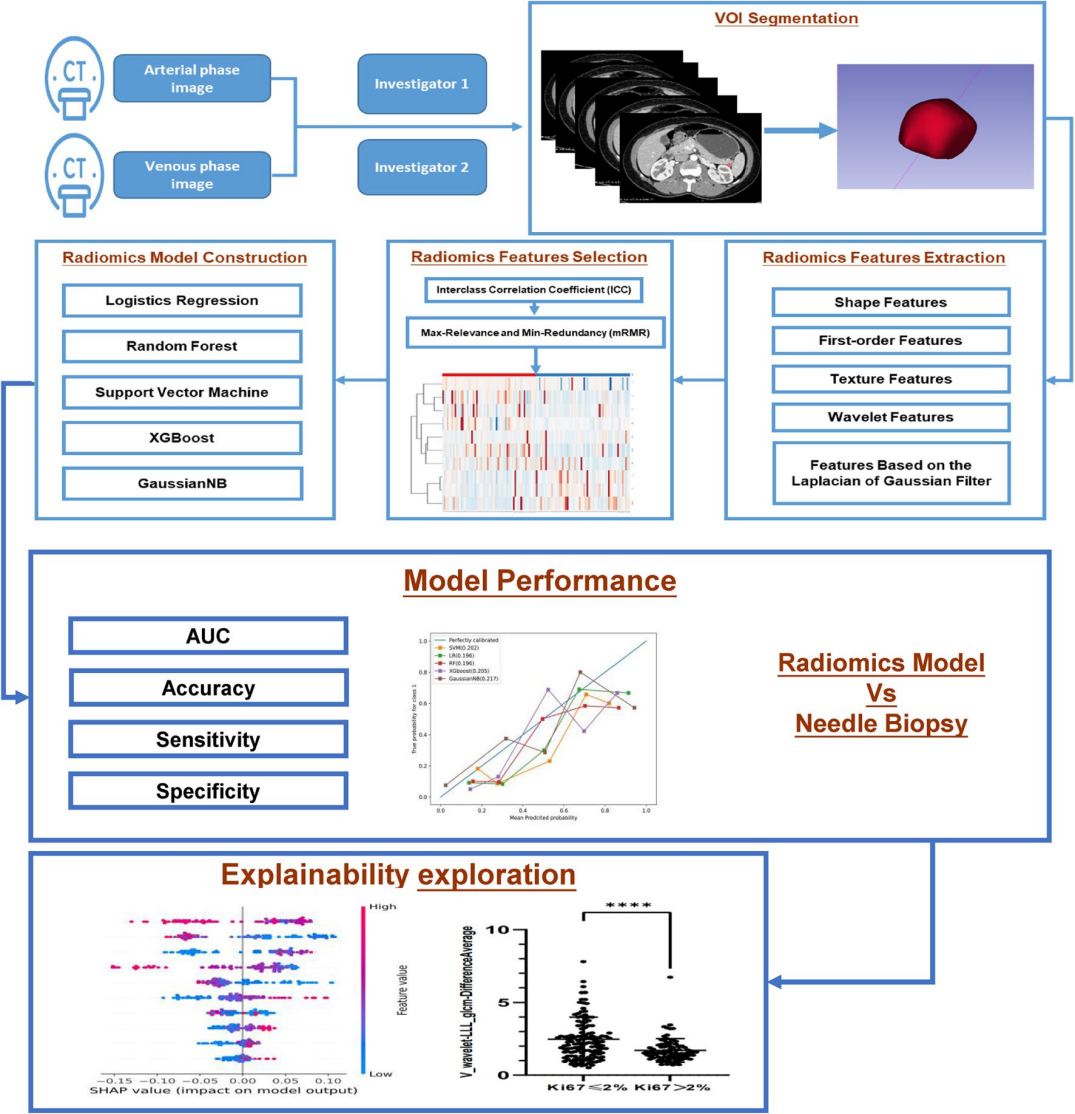
Workflow of the study

CT image acquisition was shown in Appendix E1 (Supplementary Material). Figure 2 illustrates the workflow of this study.

### Tumor segmentation

The region of interest (ROI) was segmented utilizing 3D Slicer software (version 5.0.3; www.slicer.org) layer by layer in the arterial and venous phases images by two investigators. Tumor segmentation was independent in the arterial and venous phases. Each investigator was unaware of the other’s segmentation and segmented the lesions independently. The clinical information was blinded to the investigators. The ROI defined in the study was the entire tumor area within the tumor boundary visible to the radiologists. Vessels and bile ducts were excluded from the ROI as much as possible. For multiple tumors, the tumor with the largest diameter was selected as the representative [23].

### Radiomics feature extraction and reproducibility analysis

The images were resampled, and grayscale discretized prior to feature extraction. CT images were resampled to isometric voxels of 3 × 3 × 3 mm^3^ using spline interpolation. Hounsfield units were binarized to discrete values of 25 HU. CT-based radiomics features were extracted using the Pyradiomics package [24] (version 3.0) for Python (version 3.7.3). The entire procedure conformed to the Image Biomarker Standardization Initiative guidelines [25]. A random sample of 30 CT images was used to test interobserver reproducibility for radiomics features. The interclass correlation coefficient (ICC) was applied to assess the consistency of the ROI delineation between the two investigators. Good consistency was defined as an ICC greater than 0.75. Radiomics features with ICC < 0.75 were excluded.

### Feature selection and radiomics model construction

A normalization step was performed before feature selection to eliminate data value dimensional differences. Each feature in the training set was subtracted by the mean value and divided by the standard deviation. The features in the testing set were normalized using the mean values and standard deviations calculated from the training set. The remaining significant features after reproducibility analysis were ranked using the maximum relevance minimum redundancy (mRMR) algorithm [26]. The Maximum Relevance Minimum Redundancy (mRMR) algorithm is a filter-type feature selection method widely used in complicated biological problems, particularly for feature selection in radiomics analysis. This algorithm selects a subset of features that have the highest correlation with the class (relevance) and the lowest correlation among themselves (redundancy).

Selected features were used to develop radiomics-based ML models using five ML algorithms commonly used in the biomedical field, including logistic regression (LR), GaussianNB, random forest (RF), support vector machine (SVM), and XGBoost. The hyperparameters were optimized using GridSearch with fivefold cross-validation. A threshold probability of 0.5 was applied in this study. The trained radiomics-based ML models were deployed to the time-independent testing set. The average AUC, sensitivity, specificity, accuracy, and 95%CI were calculated from 1000 bootstrap samples of the training and testing sets. The calibration curve with the Brier score loss was utilized to evaluate the calibration of models in the testing set. Ultimately, the best-performing model was selected as the final model. We compared the accuracy of the needle biopsy and the final model in assessing the pathological grade of pNETs in five patients who underwent both needle biopsy and surgical resection.

### Model interpretability exploration

The SHAP technique enables clinicians to understand the results of ML models in an explainable manner [27], which enhances model transparency by providing global and local explainability. The features are ranked in order of importance, with the features ranked higher providing a greater contribution to the model. Furthermore, the SHAP value plots can illustrate the positive and negative contributions of the features to the model. We used a summary SHAP plot to display the most prominent features in the final model.

To investigate the correlation between biomarkers and radiomics features, we compared the differences in radiomics features selected by SHAP between different Ki-67 index and mitotic count groups.

### Statistical analysis

The mean and standard deviation (SD) were utilized to describe normally distributed continuous data, and nonnormally distributed continuous data was expressed as the median (upper and lower quartiles). Categorical data were expressed as frequencies and percentages, which were compared using the chi-square test. Nonnormally distributed continuous variables were compared using the Mann–Whitney *U* test, and an independent sample *t*-test was used to compare normal-distributed continuous variables. KNNimputer algorithm was used to handle missing values in the study [28]. Accuracy was analyzed via McNemar’s test at a significance level of 0.05. All the above analyses were completed by R software (version 4.1.1; www.r-project.org) and Python software (version 3.7.3). This radiomic study was evaluated by calculating a radiomic quality score [29]. A *p*-value less than 0.05 indicated statistical significance.

## Results

### Patient characteristics

The study included 222 patients with pNETs. The training set consisted of 122 patients (mean age:50 ± 14 years; 53 male), whereas the testing set comprised 100 patients (mean age: 48 ± 13 years; 50 male). No significant differences were observed before and after the imputation of carbohydrate antigen-199, carbohydrate antigen-125, and carcinoembryonic antigen between the two sets (Appendix Table 1).

In the training set, 60 patients were diagnosed with G1 tumors and 62 patients were diagnosed with G2/G3 tumors. In the testing set, 64 patients were diagnosed with G1 tumors and 36 patients were diagnosed with G2/G3 tumors. Among the patients in the training set, 35 had functional tumors, and in the testing set, 46 patients had functional tumors. Multiple tumors were found in 23 and 16 patients in the training and testing sets, respectively (Table 1).

**Table 1:** Patients characteristics

Characteristics		Training set *N* = 122	Testing set *N* = 100
Gender (%)	Male	53 (43.4)	50 (50.0)
	Female	69 (56.6)	50 (50.0)
Age (years)		49.7 (13.5)	47.7 (13.2)
Body mass index (kg/m^2^)		22.9 (20.7,25.1)	23.5 (19.8,27.2)
Diabetes (%)	No	112 (91.8)	91 (91.0)
	Yes	10 (8.2)	9 (9.0)
Hypertension (%)	No	96 (78.7)	92 (92.0)
	Yes	26 (21.3)	8 (8.0)
Smoking (%)	No	102 (83.6)	89 (89.0)
	Yes	20 (16.4)	11 (11.0)
Drinking (%)	No	108 (88.5)	94 (94.0)
	Yes	14 (11.5)	6 (6.0)
CEA (ng/ml)		1.7 (1.1, 2.8)	1.8 (1.3, 2.7)
CA125 (U/L)		10.9 (7.8, 15.7)	11.2 (8.8, 16.2)
CA199 (U/L)		7.6 (3.6, 16.3)	8.9 (4.4, 16.4)
Tumor site (%)	Head and (or) neck	41 (33.6)	49 (49.0)
	Body and (or) tail	80 (65.6)	44 (44.0)
	Others	1 (0.8)	7 (7.0)
Multiple tumors (%)	No	99 (81.1)	83 (83.8)
	Yes	23 (18.9)	16 (16.2)
Functional tumor (%)	No	87 (71.3)	54 54.0)
	Yes	35 (28.7)	46 (46.0)
Ki-67 index (%)	≤ 2	65 (53.3)	69 (69.0)
	3–20	53 (43.4)	29 (29.0)
	> 20	4 (3.3)	2 (2.0)
Mitotic count (%)	< 2	85 (69.7)	83 (83.0)
	2–20	34 (27.8)	17 (17.0)
	> 20	3 (2.5)	0 (0.0)
Grade (%)	G1	60 (49.2)	64 (64.0)
	G2/3	62 (50.8)	36 (36.0)
AJCC stage (%)	I	41 (33.6)	42 (42.0)
	II	41 (33.6)	38 (38.0)
	III	9 (7.4)	13 (13.0)
	IV	31 (25.4)	7 (7.0)

### Feature selection

A total of 1316 features were extracted from each of the arterial and venous phase images; 747 of these had an intraclass coefficient (ICC) greater than 0.75 for the arterial phase, and 401 had an ICC greater than 0.75 for the venous phase (Appendix Fig. 1). Out of the extracted features, 1148 were input into the maximum relevance minimum redundancy (mRMR) algorithm for feature selection. The 10 most relevant features selected by the algorithm are listed in Appendix E2. The differences in these radiomics features between G1 and G2/3 tumors were demonstrated using a heatmap (Appendix Fig. 2).

### Development and testing of radiomics-based ML model

**Fig. 3:**
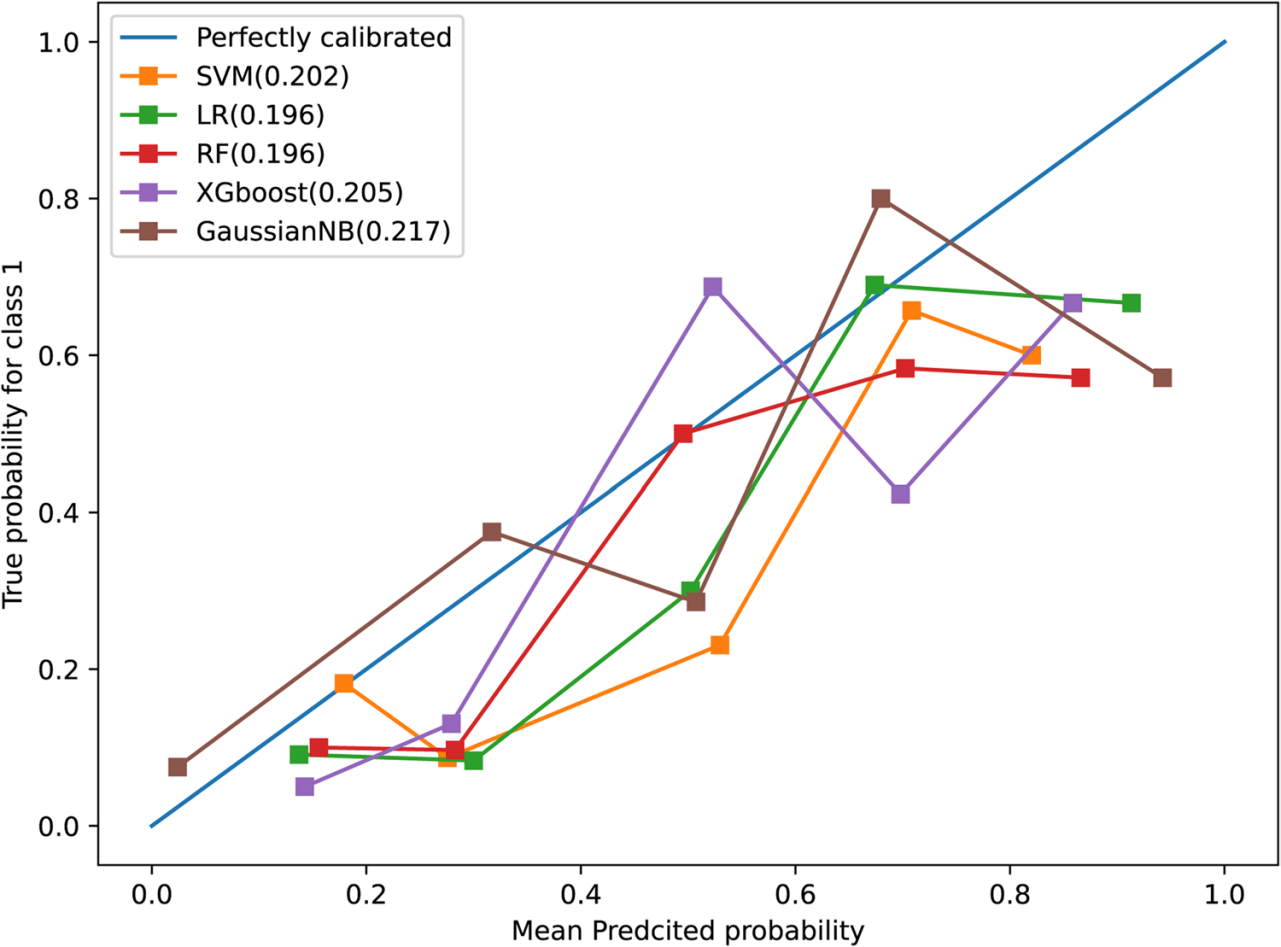
Calibration curves of five radiomics-based machine learning models in the testing set. The Brier score loss of each model is shown in brackets

The hyperparameters for each model used in the study are shown in Appendix E3. In the training set, the average AUC values for the models were 0.727 (95%CI: 0.725, 0.730) for LR, 0.827 (95%CI: 0.825, 0.829) for RF, 0.769 (95%CI: 0.767, 0.772) for SVM, 0.811 (95%CI: 0.809, 0.813) for XGBoost, and 0.705 (95%CI: 0.703, 0.708) for GaussianNB. The average sensitivity, specificity, and accuracy values are presented in Table 2. In the testing set, the AUC values were 0.758 (95%CI: 0.756, 0.761) for LR, 0.742 (95%CI: 0.740, 0.745) for SVM, 0.779 (95%CI: 0.776, 0.782) for RF, 0.744 (95%CI: 0.742, 0.747) for XGBoost, and 0.745 (95%CI: 0.742, 0.748) for GaussianNB. The accuracy values were 73.0% (95%CI: 72.7%, 73.3%) for LR, 70.0% (95%CI: 69.8%, 70.3%) for SVM, 77.0% (95%CI, 76.8%, 77.3%) for RF, 71.9% (95%CI: 71.7%, 72.2%) for XGBoost, and 72.9% (95%CI: 72.6%, 73.1%) for GaussianNB (Table 3). The calibration curves for the five models are shown in Fig. 3. The Brier score loss was 0.196 for LR, 0.202 for SVM, 0.196 for RF, 0.205 for XGBoost, and 0.217 for GaussianNB. The RF model was selected as the final model owing to its higher AUC and accuracy and lower Brier score loss. However, compared to biopsy results (accuracy: 40%, 2 of 5 patients), the final model achieved an accuracy of 80% (4 of 5 patients) in evaluating the pathological grade of pNETs; however, this difference was not statistically significant (*p* = 0.125, Appendix Table 2). The radiomics quality score of the study was 15, suggesting a favorable quality (Appendix E4).

**Table 2:** Diagnostic performance of the radiomics-based machine learning models in the training cohort

	AUC (95%CI)	Sensitivity (95%CI)	Specificity (95%CI)	Accuracy (95%CI)
LR (%)	0.727 (0.725, 0.730)	80.2 (79.8, 80.5)	65.3 (65.0, 65.7)	72.7 (72.5, 72.7)
SVM (%)	0.769 (0.767, 0.772)	86.8 (86.5, 87.0)	67.1 (66.8, 67.5)	76.9 (76.7, 77.2)
RF (%)	0.827 (0.825, 0.829)	87.4 (83.5, 91.3)	84.2 (82.2, 86.2)	82.7 (82.5, 82.9)
XGBoost (%)	0.811 (0.809, 0.813)	82.2 (79.6, 84.8)	81.4 (79.2, 83.6)	81.1 (80.9, 81.3)
GaussianNB (%)	0.705 (0.703, 0.708)	73.7 (73.3, 74.0)	67.4 (67.0, 67.7)	70.5 (70.3, 70.8)

*LR* logistic regression, *SVM* support vector machine, *RF* random forest, *CI* confidence interval

**Table 3:** Diagnostic performance of the radiomics-based machine learning models in the time-independent testing cohort

	AUC (95%CI)	Sensitivity (95%CI)	Specificity (95%CI)	Accuracy (95%CI)
LR (%)	0.758 (0.756, 0.761)	86.0 (85.7, 86.4)	65.6 (65.3, 66.0)	73.0 (72.7, 73.3)
SVM (%)	0.742 (0.740, 0.745)	89.2 (88.8, 89.5)	59.3 (59.0, 59.7)	70.0 (69.8, 70.3)
RF (%)	0.779 (0.776, 0.782)	80.8 (80.4, 81.3)	74.9 (74.6, 75.3)	77.0 (76.8, 77.3)
XGBoost (%)	0.744 (0.742, 0.747)	83.2 (82.8, 83.6)	65.6 (65.3, 66.0)	71.9 (71.7, 72.2)
GaussianNB (%)	0.745 (0.742, 0.748)	80.4 (80.0, 80.8)	68.7 (68.3, 69.0)	72.9 (72.6, 73.1)

*LR* logistic regression, *SVM* support vector machine, *RF* random forest, *CI* confidence interval

To investigate whether different scanners have an impact on the predictive power of the model, we predicted the pathological grade of pNETs among patients in the test set who were examined using different scanners. The results showed no statistical difference in the AUC between the two scanners (0.782 vs. 0.758, *p* = 0.36, DeLong test). This indicated that the predictive power of the model remained consistent across scanners, suggesting that inter-scanner variability did not significantly influence the results.

We explored independent predictors of the pathological grade of pNETs based on clinical characteristics using univariable and multivariable logistic regression in clinical characteristics. The results suggested that tumor size and CT-reported liver metastases were independent predictors of pathological grade (Appendix Table 3). Accordingly, a clinical model was developed utilizing multivariable logistic regression, incorporating tumor size and CT-reported liver metastases as key factors. Furthermore, we generated predicted probabilities, referred to as Radscore, from the radiomics model and integrated them with the clinical model to establish a combined model. Appendix Table 4 presents the AUC values for the radiomics model, clinical model, and combined model in the testing set. The results demonstrated a statistically significant distinction difference in the AUC values between the clinical and radiomics models (AUC: 0.791 vs. 0.711, *p* = 0.03). However, the difference between the combined model and the radiomics model was not statistically significant (AUC: 0.791 vs. 0.788, *p* = 0.81).

### Exploration of model interpretability

**Fig. 4:**
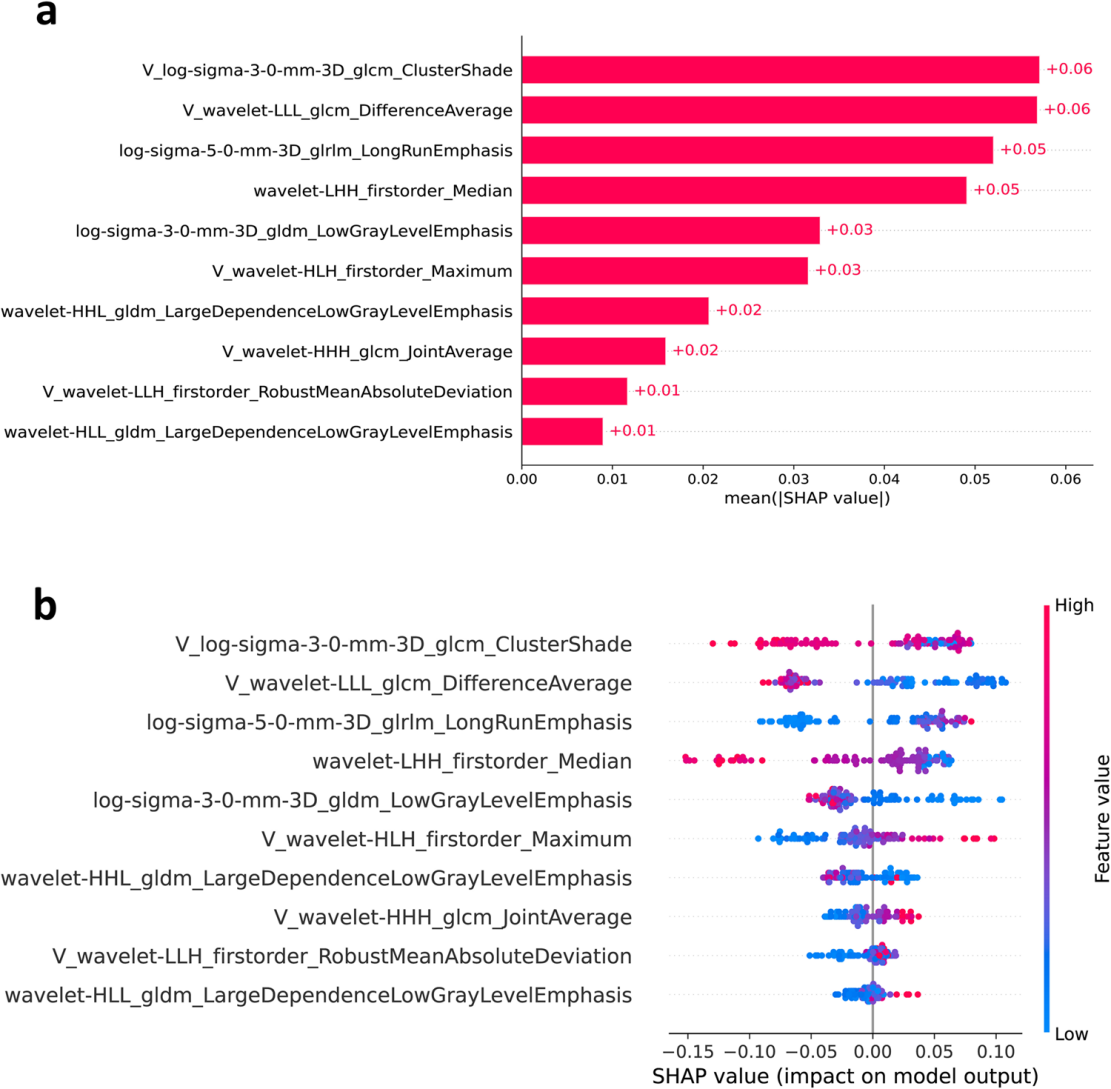
SHAP summary plot. **a** Ranking the importance of each feature in the final model output. **b** SHAP values of each feature in the final model. The different colors (red and blue) represent different levels of effect on the output of the model

**Fig. 5:**
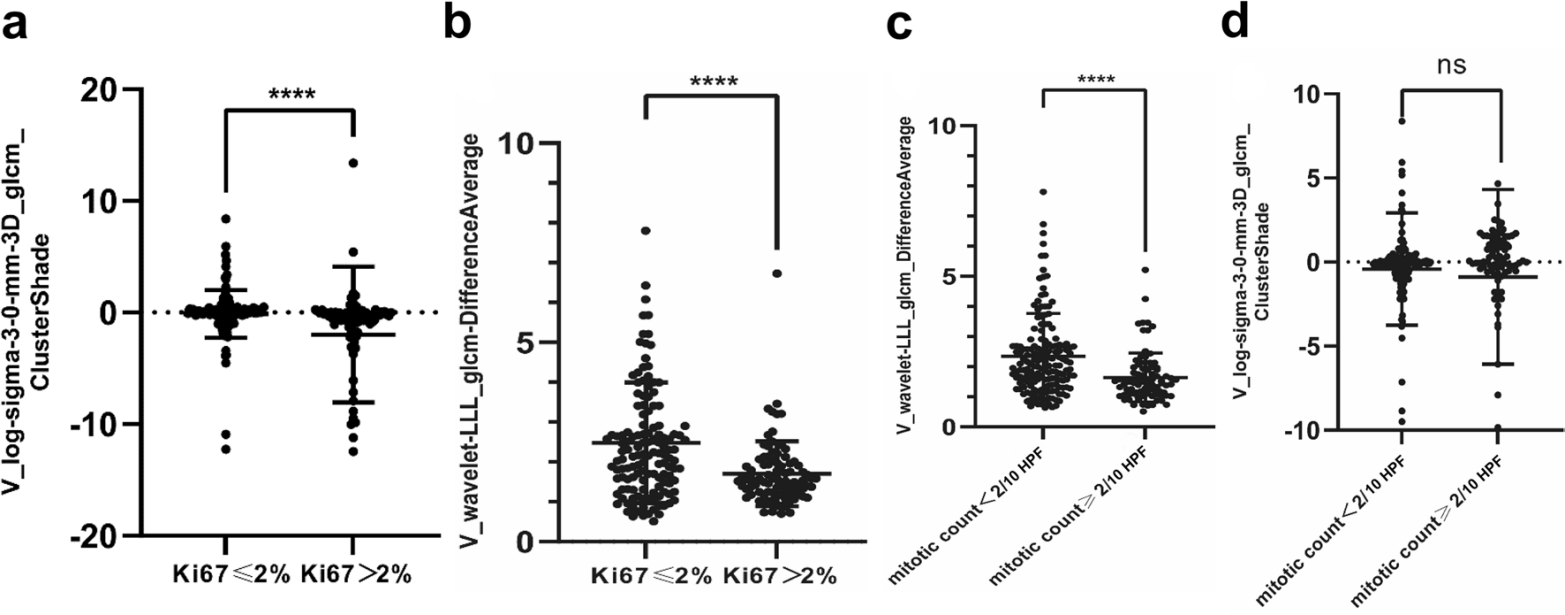
The relationship between biological and radiomics features. **a** Comparison of V_log-sigma-3–0-mm-3D_glcm_ClusterShade feature between different Ki-67 index groups. **b** Comparison of V_wavelet-LLL_glcm_DifferenceAverage feature between different Ki-67 index groups. **c** Comparison of V_log-sigma-3–0-mm-3D_glcm_ClusterShade feature between different mitotic count groups. **d** Comparison of V_wavelet-LLL_glcm_DifferenceAverage feature between different mitotic count groups. V_, venous phase; HPF, high power field; ns, no significance. ****p < 0.001

As illustrated in Fig. 4, wavelet-LLL_glcm_DifferenceAverage (venous phase) and log-sigma-3–0-mm-3D_glcm_ClusterShade (venous phase) were the top features that contributed the most to the model. The log-sigma-3–0-mm-3D_glcm_ClusterShade (venous phase) feature was significantly different between the Ki-67 ≤ 2% and Ki-67 > 2% groups (*p* < 0.001), but not between the mitotic count < 2/10 high power field (HPF) and mitotic count ≥ 2/10 HPF groups. Significant differences were observed in the wavelet-LLL_glcm_DifferenceAverage (venous phase) feature between the two groups with Ki-67 ≤ 2% and Ki-67 > 2% (*p* < 0.001), as well as between the two groups with mitotic count < 2/10 HPF and mitotic count ≥ 2/10 HPF (*p* < 0.001; Fig. 5).

## Discussion

In this study, an interpretable ML model was constructed and tested to differentiate between G1 and G2/3 of pNETs. The model was developed by analyzing radiomic features extracted from contrast-enhanced CT images obtained during the arterial and venous phases. In the testing set, the radiomics-based ML model demonstrated favorable discrimination (AUC, 0.779; accuracy, 77.0%) and calibration. The model also exhibited promising interpretability, allowing for a better understanding of the factors contributing to its predictions.

Previous studies have highlighted the significant potential of texture analysis and radiomics in predicting the pathological grade of pNETs [30, 31]. A study involving 137 patients with pNETs developed a nomogram model combining radiomics features derived from arterial phase images and clinical features to classify G1 and G2/3 tumors [32]. The AUCs of the training and validation sets were 0.907 and 0.891, respectively, indicating that the model showed good discrimination capability, along with favorable calibration and clinical usefulness. Bian et al also developed a CT radiomics score that showed a significant association with the grade of non-functional pNETs (NF-pNETs) and provided a potentially valuable non-invasive tool for differentiating between different grades of NF-pNETs, especially in patients with tumors measuring 2 cm or less [33]. With the advancement of artificial intelligence, the combination of radiomics analysis and ML algorithms has further enhanced the predictive power of ML-based models. ML algorithms can be applied to feature selection, model construction, or both. In a previous study involving 138 patients with pNETs, mRMR and RF algorithms were utilized for radiomics feature selection and model development for predicting G1 and G2/3 tumors [18], resulting in a nomogram that integrated tumor margin and radiomics signature. This nomogram showed strong discrimination with AUCs of 0.974 in the training set and 0.902 in the validation set. Zhang et al [17] constructed diagnostic models for classifying the pathological grades of pNETs by integrating five methods for feature selection and nine methods for classification. Likewise, all the radiomics-based ML models presented in this study had AUCs greater than 0.74 and an accuracy greater than 0.7, confirming the great promise of radiomics analysis combined with ML in predicting the pathological grade of pNETs. Although the accuracy of the model developed in this study did not exceed that of certain existing studies, its noteworthy contribution lies in its interpretability and the direct comparison between the accuracy of radiomics models and needle biopsy.

**Fig. 6:**
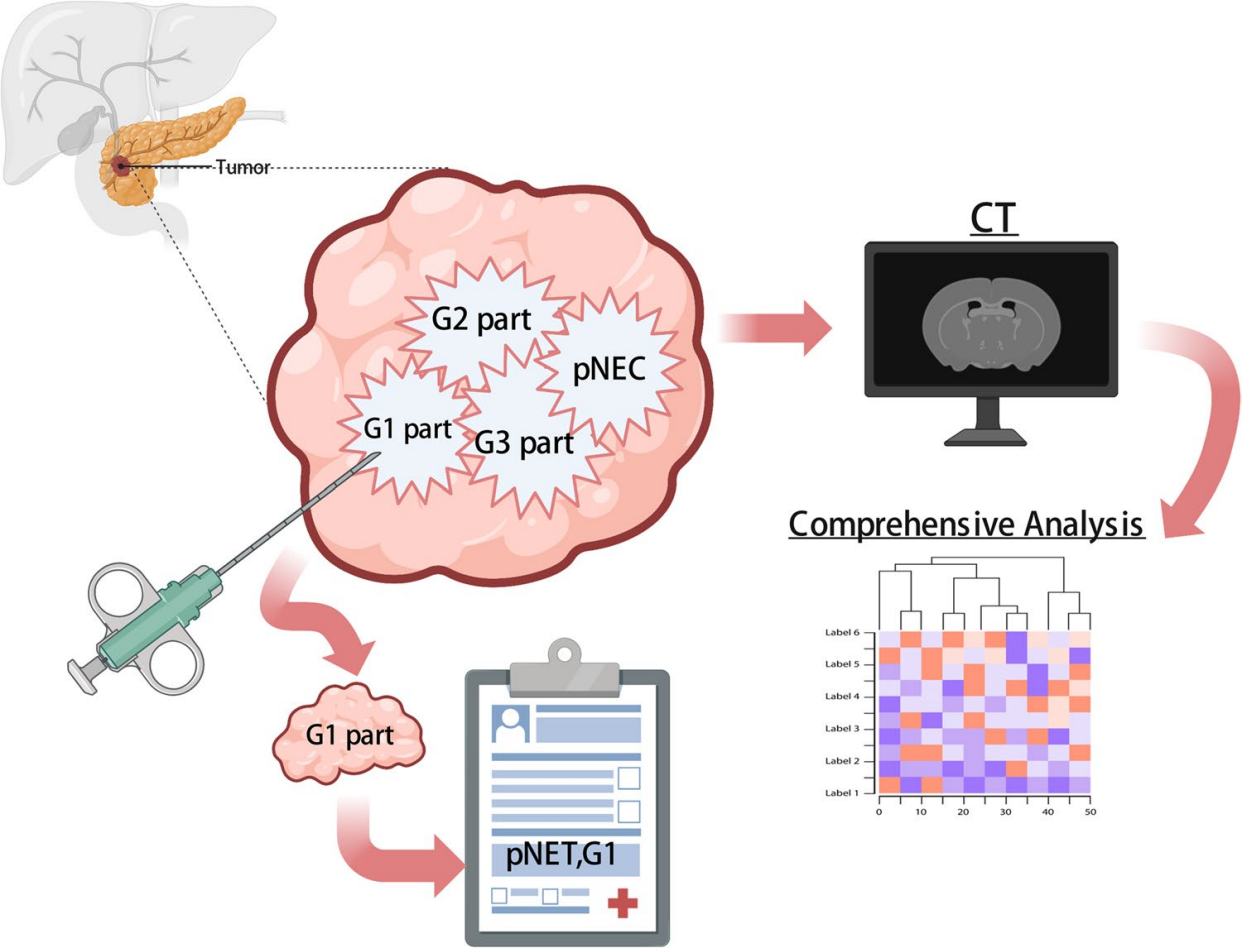
Comparison of needle biopsy and radiomics analysis in the presence of intratumoral heterogeneity of pNETs. Due to the random nature of biopsy sampling and the limited samples, biopsy results may not be representative of the entire tumor. In contrast, radiomics analysis can provide comprehensive information that encompasses the entire tumor (created with BioRender.com)

Notably, this study compared the accuracy of radiomics models and needle biopsy results in the evaluation of the pathological grade of pNETs in a small sample. Although the results of the comparison did not lead to a definitive conclusion, they served as an important reminder. pNETs can show significant heterogeneity between patients and within tumor tissue, which is defined as spatial intratumoral heterogeneity [34, 35]. Proliferative heterogeneity is an essential manifestation of this intratumoral heterogeneity. Tang et al evaluated 31 well-differentiated NETs with indications of a high-grade tumor composition [36]. Heterogeneity in the Ki-67 index was also observed in both primary tumors and liver metastases of NETs [37]. Therefore, the pNETs tumor tissue may contain several components with different proliferative rates. Due to the random selection of the biopsy sites and the limited size of the tissue obtained, it is possible that needle biopsy results do not accurately reflect the proliferative status of the entire tumor (Fig. 6). Although previous studies have reported that the coincidence rates of pathological grade assessed using the mean and highest Ki-67 index in the specimens obtained by EUS-FNA with the resected specimens were 74.0% and 77.8% [38], the invasive nature of needle biopsy and the risk of puncture failure should not be ignored. In contrast, radiomics analysis based on non-invasive imaging can provide high-throughput and more comprehensive information about the tumor. By analyzing the tumor as a whole, radiomics analysis may help to resolve some of the challenges associated with the pathological examination of small or random biopsy samples of heterogeneous tumors, serving as a comprehensive “virtual biopsy” [39], which is gaining recognition in the era of advances in imaging technology and is considered a reliable complement to traditional tumor biopsy [40].

Although previous studies have reported excellent performance of radiomics-based ML models for preoperative prediction of the pathological grade of pNETs, few models have been applied to clinical practice, and several factors contribute to this limitation. Firstly, the small sample sizes reduced the credibility of the results, with no study exceeding 200 cases. Secondly, the lack of exploration of model interpretability has resulted in a “black-box” effect, which hampers clinical application [41]. Thirdly, current studies have not established the correlation between radiomics features and biomarkers in pNETs. In addition, the inconsistency in the selection of radiomics features across different studies undermines the trust in these models. Thus, establishing a link between radiomics features and biomarkers is essential for the promotion of these models.

To address these challenges, this study attempted to answer questions regarding model interpretability and explainability. According to previous studies [42], interpretability is the degree to which a human can understand or intuit how a model has reached a specific outcome, whereas explainability refers to the internal mechanisms and the logic of the ML system. As a first step, we used the SHAP tool to identify the two most influential gray-level co-occurrence matrix (GLCM) features of the venous phase, which demonstrated the explainability of the model. GLCM features assess the texture of an image based on spatial alignment statistics of pixel intensity and are known to be common and sensitive texture descriptors [43]. A previous study reported that GLCM features provide information about tumor heterogeneity and reflect prognostic changes in gastrointestinal stromal tumors [44]. The “cluster shade” of GLCM features can be interpreted as a measure of skewness, reflecting the presence of large variation and high grayscale levels. Additionally, GLCM features can characterize the contrast, intricacy, and heterogeneity of local strength modes, potentially indicating the proliferative heterogeneity of pNETs. Furthermore, we believed that annotation of radiomics features by establishing relationships between radiomics features and known biological features in specific diseases is a viable option for exploring the biological significance of radiomics features. Therefore, in this study, we found that these two features showed significant differences among different groups based on the Ki-67 index or mitotic count, demonstrating the interpretability of the model. In a radiomic–genomic study of lung cancer, researchers found that different radiomic features were correlated with diverse biological processes. It was shown that texture entropy and clustering features, as well as voxel intensity differences, were related to immune function, the status of the P53 pathway, and other pathways involved in cell cycle regulation [45]. These findings suggest that radiomics features can characterize the underlying biological processes. Establishing the association between radiomics features and biomarkers can enhance the accessibility of radiomics-based models for clinicians, as causal inference plays a vital role in the biomedical field.

It is important to highlight that this study incorporated two filters, resulting in the generation of a substantial number of filter-transformed features. Dealing with such a large number of higher-order features poses the challenge of eliminating redundant features while retaining the most informative ones. To address this, Benedetti et al [46] employed a correlation-based filter and identified independent and informative radiomics features based on the AUC value, enabling further analysis.

In this study, to effectively reduce feature dimensionality, we initially used interclass correlation coefficients to select features with favorable reproducibility. Subsequently, the mRMR algorithm was applied for feature selection. The mRMR algorithm considers feature correlations comprehensively during the selection process, ensuring the exclusion of highly correlated features to maintain model simplicity. Another important issue that requires attention is the statistical challenge arising from the substantial increase in features. Specifically, the notable augmentation of high-order features can render the filter-transformed features more susceptible to random selection. Consequently, the judicious utilization of statistically rigorous methods during the feature selection process becomes paramount to mitigate this selective bias in radiomics. However, it is worth noting that there is currently no standardized procedure for feature selection. Therefore, it becomes particularly crucial to explore the interpretability of the selected features in such cases. In our study, we established correlations between higher-order features and significant clinicopathologic features, providing substantial evidence that the features incorporated in the model were not selected by chance.

This study has several limitations that should be acknowledged. Firstly, the data used in this study were obtained exclusively from one of the two Canon CT scanners at a single medical center. Therefore, it is important to corroborate the results of this investigation through a prospective cohort study that encompasses multiple centers to ensure the generalizability of the results. Furthermore, owing to the limited sample size of patients who both underwent preoperative biopsy and surgical resection, this study only compared the accuracy of radiomics models and biopsy. In the future, a further study with a larger sample size could be conducted to compare the AUC, sensitivity, and specificity of these two methods. Finally, this study only preliminarily explored the relationship between radiomics features and biomarkers in predicting the pathological grade of pNETs. Further research can be conducted by combining radiomics–genomics and pathomics analyses to delve deeper into the biological significance of radiomics features.

In conclusion, the radiomics-based interpretable machine learning model holds promise for predicting the pathological grade in pNETs and establishing a link between radiomics features and biomarkers. As a preoperative assessment tool for the pathological grade of pNETs, radiomics models could provide a crucial supplementary approach to preoperative needle biopsy.

### Supplementary Information

Below is the link to the electronic supplementary material.

Supplementary file1 (PDF 392 KB)

## Abbreviations

AUC: Area under the curve
EUS-FNA: Endoscopic ultrasound-guided fine needle aspiration
GLCM: Gray-level co-occurrence matrix
HPF: High power field
ICC: Interclass correlation coefficient
LR: Logistic regression
ML: Machine learning
mRMR: Maximum relevance minimum redundancy
NCCN: National Comprehensive Cancer Network
pNETs: Pancreatic neuroendocrine tumors
RF: Random forest
SD: Standard deviation
SHAP: Shapley Additive Explanation
SVM: Support vector machine

## Declarations

### Guarantor

The scientific guarantor of this publication is Xiao-Yu Yin.

### Statistics and biometry

No complex statistical methods were necessary for this paper.

### Study subjects or cohorts overlap

There was no study subjects or cohorts overlap in this study.

### Methodology

• retrospective

• diagnostic or prognostic study

• performed at one institution
