# Supplementary material for: A radiomics-based interpretable model to predict the pathological grade of pancreatic neuroendocrine tumors
Source: Eur Radiol. 2023 Sep 2;34(3):1994–2005. doi: 10.1007/s00330-023-10186-1 (PMC10873440; doi:10.1007/s00330-023-10186-1)

## Supplemental Materials

### Appendix E1 CT image acquisition

All contrast-enhanced CT images were obtained using 80-multidetector spiral CT scanner (Aquilion 80, Canon Medical) or 320-multidetector spiral CT scanner (Aquilion 320, Canon Medical). The scanning parameters were as follows: 120 kV tube voltage, 250 mA tube current, 0.5 mm scanning layer thickness and layer spacing, 1.0 pitch, 512×512 matrix. After aroutine unenhanced scan, 1.5 mL/kg of contrast media (Ultravist, Bayer) was injected into an antecubital vein at a rate of 3.0-4.0 mL/s via a pump injector (P3Tabdomen module, Medrad Inc.). Arterial scanning was performed at 35 s, and venous scanning was performed at 65 s after injection. The original scanned data were reconstructed with a layer thickness and interval of 1.0 mm.

### Appendix E2 Radiomics features selected by MRMR algorithm

wavelet-LLL_glcm_DifferenceAverage (venous phase)
wavelet-HHH_glcm_JointAverage (venous phase)
wavelet-HHL_gldm_LargeDependenceLowGrayLevelEmphasis (arterial phase)
log-sigma-5-0-mm-3D_glrlm_LongRunEmphasis (arterial phase)
log-sigma-3-0-mm-3D_gldm_LowGrayLevelEmphasis (arterial phase)
wavelet-LHH_firstorder_Median (arterial phase)
log-sigma-3-0-mm-3D_glcm_ClusterShade (venous phase)
wavelet-LLH_firstorder_RobustMeanAbsoluteDeviation (arterial phase)
wavelet-HLL_gldm_LargeDependenceLowGrayLevelEmphasis (arterial phase)
wavelet-HLH_firstorder_Maximum (venous phase)

### Appendix E3 The hyperparameters of each machine learning model

**1. LR**

penalty='l2', dual=False, tol=0.0001, C=1.0, fit_intercept=True, intercept_scaling=1, class_weight=None, random_state=0, solver='lbfgs', max_iter=100, multi_class='auto', verbose=0, warm_start=False, n_jobs=None, l1_ratio=None.

**2. SVM**

C=1.0, kernel='rbf', degree=3, gamma='0.1', coef0=0.0, shrinking=True, probability=True, tol=0.001, cache_size=200, class_weight=None, verbose=False, max_iter=-1, decision_function_shape='ovr', break_ties=False, random_state=0.

**3. RF**

n_estimators=37, criterion='gini', max_depth=3, min_samples_split=2, min_samples_leaf=1, min_weight_fraction_leaf=0.0, max_features=3, max_leaf_nodes=None, min_impurity_decrease=0.0, bootstrap=True, oob_score=False, n_jobs=None, random_state=0, verbose=0, warm_start=False, class_weight=None, ccp_alpha=0.0, max_samples=None.

**4. XGBoost**

learning_rate =0.05, n_estimators=100, max_depth=3, min_child_weight=5, gamma=0.3, subsample=0.6, colsample_bytree=0.6, objective= 'binary:logistic', nthread=4, reg_lambda=1, reg_alpha=1e-05, scale_pos_weight=1, random_state=0.

**5. GaussianNB**

Default parameters

**Appendix E4 The radiomics quality score of this study**

Image protocol quality - well-documented image protocols (for example, contrast, slice thickness, energy, etc.) and/or usage of public image protocols allow reproducibility/replicability

√protocols well documented

public protocol used

none

Multiple segmentations - possible actions are: segmentation by different physicians/algorithms/software, perturbing segmentations by (random) noise, segmentation at different breathing cycles. Analyse feature robustness to segmentation variabilities

√yes

no

Phantom study on all scanners - detect inter-scanner differences and vendor-dependent features. Analyse feature robustness to these sources of variability

yes

√no

Imaging at multiple time points - collect images of individuals at additional time points. Analyse feature robustness to temporal variabilities (for example, organ movement, organ expansion/shrinkage)

yes

√no

Feature reduction or adjustment for multiple testing - decreases the risk of overfitting. Overfitting is inevitable if the number of features exceeds the number of samples. Consider feature robustness when selecting features

√Either measure is implemented

Neither measure is implemented

Multivariable analysis with non radiomics features (for example, EGFR mutation) - is expected to provide a more holistic model. Permits correlating/inferencing between radiomics and non radiomics features

yes

√no

Detect and discuss biological correlates - demonstration of phenotypic differences (possibly associated with underlying gene–protein expression patterns) deepens understanding of radiomics and biology

√yes

no

Cut-off analyses - determine risk groups by either the median, a previously published cutoff or report a continuous risk variable. Reduces the risk of reporting overly optimistic results

yes

√no

Discrimination statistics - report discrimination statistics (for example, C-statistic, ROC curve, AUC) and their statistical significance (for example, p-values, confidence intervals).

One can also apply resampling method (for example, bootstrapping, cross-validation)

√a discrimination statistic and its statistical significance are reported

a resampling method technique is also applied

none

Calibration statistics - report calibration statistics (for example, Calibration-in-thelarge/slope, calibration plots) and their statistical significance (for example, P-values, confidence intervals). One can also apply resampling method (for example, bootstrapping, cross-validation)

√a calibration statistic and its statistical significance are reported

a resampling method technique is applied

none

Prospective study registered in a trial database - provides the highest level of evidence supporting the clinical validity and usefulness of the radiomics biomarker

yes

√no

Validation - the validation is performed without retraining and without adaptation of the cut-off value, provides crucial information with regard to credible clinical performance

No validation

√validation is based on a dataset from the same institute

validation is based on a dataset from another institute

validation is based on two datasets from two distinct institutes

the study validates a previously published signature

validation is based on three or more datasets from distinct institutes

Comparison to 'gold standard' - assess the extent to which the model agrees with/is superior to the current 'gold standard' method (for example, TNM-staging for survival prediction). This comparison shows the added value of radiomics

√yes

no

Potential clinical utility - report on the current and potential application of the model in a clinical setting (for example, decision curve analysis).

yes

no

Cost-effectiveness analysis - report on the cost-effectiveness of the clinical application (for example, QALYs generated)

√yes

no

Open science and data - make code and data publicly available. Open science facilitates knowledge transfer and reproducibility of the study

scans are open source

region of interest segmentations are open source

the code is open sourced

√radiomics features are calculated on a set of representative ROIs and the calculated features and representative ROIs are open source

Total score

15
(41.67%)

**Appendix Table 1** The difference of CA199, CA125, CEA before and after missing value imputation

| | | **median [IQR]** | ***P* -value** |
|---|---|---|---|
| **CEA** | Before imputation | 1.7 [1.1, 2.8] | 0.961 |
| | After imputation | 1.7 [1.1, 2.8] | |
| **CA125** | Before imputation | 11.1 [8.1, 15.9] | 0.960 |
| | After imputation | 11.1 [8.1, 15.9] | |
| **CA199** | Before imputation | 8.2 [4.0, 16.3] | 1.000 |
| | After imputation | 8.2 [4.0, 16.3] | |

**Appendix Table 2** Clinico-pathological characteristics, Biopsy-obtained and model-predicted pathological grade in patients undergoing both preoperative biopsy and surgical resection

| | Gender | Age (years old) | Tumor Site | Tumor Size(cm) | Multiple Tumors | Functional Tumor | Biopsy Method | Grade from Surgical Specimen | Grade from Biopsy | Grade Predicted by Radiomics Model |
|---|---|---|---|---|---|---|---|---|---|---|
| **Patient 1** | Female | 30 | Tail | 4.6 | Yes | No | EUS-FNA | G2 | G1 | G2/3 |
| **Patient 2** | Female | 42 | Head | 2.4 | Yes | Yes | US-FNA | G2 | G1 | G2/3 |
| **Patient 3** | Female | 65 | Body and tail | 5.0 | Yes | No | US-FNA | G2 | G2 | G2/3 |
| **Patient 4** | Female | 47 | Body and tail | 4.2 | No | No | US-FNA | G2 | G1 | G2/3 |
| **Patient 5** | Male | 40 | Neck | 4.3 | No | No | US-FNA | G1 | G1 | G2/3 |

**Appendix Table 3** Univariable and multivariable logistic regression for clinical features

| Characteristics | Univariable logistic regression | | | Multivariable logistic regression | | |
|---|---|---|---|---|---|---|
| | OR | 95%CI | *P* value | OR | 95%CI | *P* value |
| **Gender** | 1.83 | 0.89-3.82 | 0.10 | | | |
| **CEA** | 0.98 | 0.88-1.03 | 0.49 | | | |
| **CA125** | 1.02 | 1.00-1.07 | 0.16 | | | |
| **CA199** | 1.00 | 0.99-1.00 | 0.48 | | | |
| **CT-reported lymph node involvement** | 2.23 | 0.92-5.71 | 0.08 | | | |
| **CT-reported liver metastases** | 8.32 | 3.14-26.40 | <0.001 | 5.25 | 1.71-18.80 | 0.006 |
| **CT-reported vascular invasion** | 5.46 | 2.02-17.50 | 0.002 | 1.33 | 0.37-5.08 | 0.70 |
| **Tumor site** | 1.52 | 0.73-3.21 | 0.27 | | | |
| **Tumor size** | 1.61 | 1.29-2.07 | <0.001 | 1.44 | 1.16-1.86 | 0.002 |
| **Multiple tumor** | 0.90 | 0.36-2.24 | 0.82 | | | |
| **Functional tumor** | 0.48 | 0.21-1.06 | 0.07 | | | |

**Appendix Table 4** Comparison of AUC values of different models

| | **AUC** | **95%CI** | ***P* value** |
|---|---|---|---|
| **Clinical model** | 0.711 | 0.608-0.813 | 0.03* |
| **Radiomics model** | 0.791 | 0.698-0.883 | |
| **Combined model** | 0.788 | 0.696-0.879 | 0.81# |

* Comparison between the clinical model and radiomics model

# Comparison between the combined model and radiomics model

**Appendix Figure 1.** Interclass correlation coefficients of (A) arterial phase features and (B) venous phase features

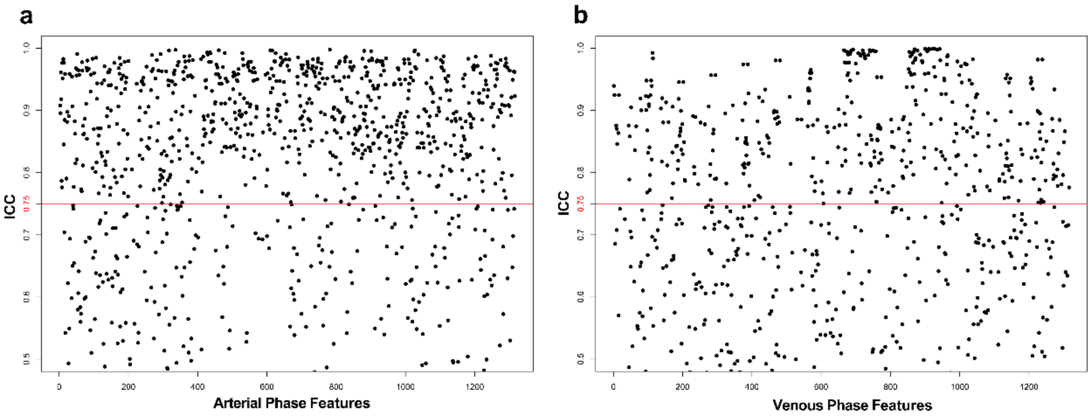

**Appendix Figure 2.** Heatmap of the 10 most relevant radiomics features for differentiation between G1 and G2/3 pNETs

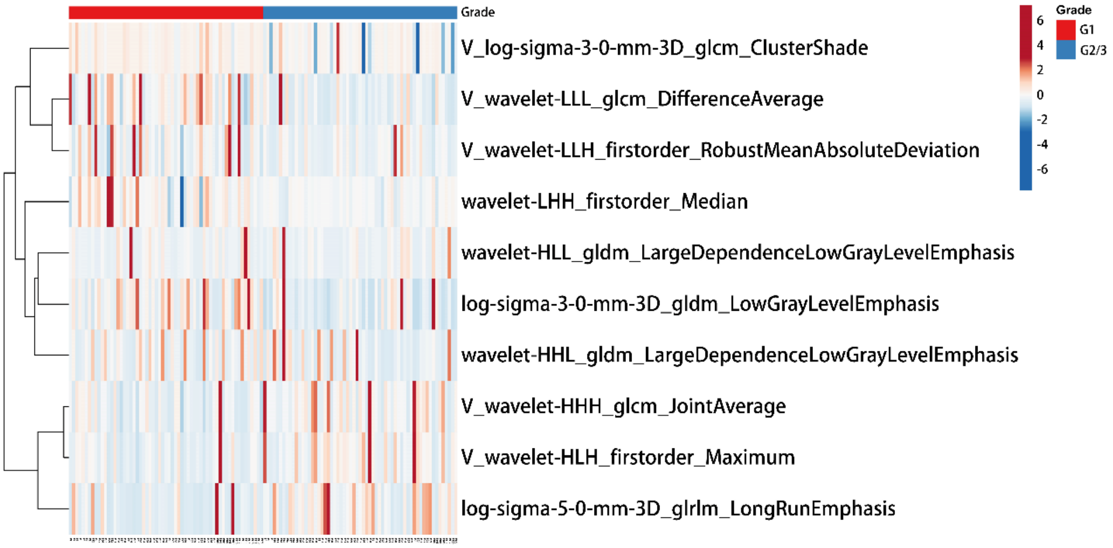

Supplement: Supplementary file 1 — Supplementary file1 (PDF 392 KB) [file 330_2023_10186_MOESM1_ESM.pdf]
